# Clinical Significance of Tumor Invasion Gene Profiling in Early‐Stage Hormone Receptor‐Positive Breast Cancer: A Cross‐Sectional Study

**DOI:** 10.1002/hsr2.72780

**Published:** 2026-07-06

**Authors:** Shayan Forghani, Hamid Rezvani, Arman Forghani, Fatemeh Mahdavi Sabet, Atieh Akbari, Sanaz Tabarestani, Hamid Reza Mirzaee

**Affiliations:** ^1^ Cancer Research Center Shahid Beheshti University of Medical Sciences Tehran Iran; ^2^ Department of Hematology‐oncology Shahid Beheshti University of Medical Sciences Tehran Iran

**Keywords:** breast cancer, CTSV, gene expression, metastasis, MYBL2, SCUBE2

## Abstract

**Background and Aims:**

Advanced breast cancer is a leading cause of cancer‐associated death in women. It is critical to find biomarkers to identify patients who are more prone to progress to advanced disease and would be better served with more aggressive treatments. Hormone receptor‐positive breast cancers make up about 70% of all breast cancers. The analysis of gene amplification and expression has been utilized to classify breast tumors into medically useful risk groups. In the present study, we selected five genes (*MMP11*, *CTSV*, *CD68*, *SCUBE2*, and *MYBL2*) that are important in tumor invasion and metastasis to identify a pattern associated with well‐established pathology markers indicative of poor prognosis in early‐stage hormone receptor‐positive breast cancers.

**Methods:**

Two hundred and thirty‐nine early‐stage hormone receptor‐positive breast cancer patients were analyzed. Patients were required to have FFPE (formalin‐fixed paraffin‐embedded) samples from the primary tumor. Expression of the selected genes was determined using TaqMan expression assays. *MYBL2* amplification was examined using fluorescent in situ hybridization (FISH).

**Results:**

Expression of *CTSV* was significantly associated with increasing tumor size (*p* = 0.001) and lymph node involvement (*p* = 0.016). In multivariate analysis, *CTSV* expression remained significantly associated with lymph node involvement (*p* = 0.049). Expression of *MYBL2* was associated with higher tumor grade (*p* = 0.001). We identified *MYBL2* amplification in one sample. Expression of *SCUBE2* was inversely associated with tumor grade (*p* = 0.04). Receiver‐operator characteristic (ROC) analysis was used to predict lymph node involvement using clinicopathologic features (age at diagnosis, tumor size, and histologic grade), and the five gene expression markers, which resulted in AUC = 0.83.

**Conclusion:**

We found a significant association between *CTSV*, *MYBL2*, and *SCUBE2* expression and pathological variables of poor prognosis. Our study reveals that analysis of the expression of these genes may help identify early‐stage hormone receptor‐positive breast tumors that are more likely to metastasize and need more intense treatment modalities.

## 1 Introduction

Globally, breast cancer is the leading malignancy diagnosed among women. Breast cancer is a heterogeneous disease, and multiple subtypes have been identified. Hormone receptor‐positive breast cancers, which express either estrogen receptor (ER) or progesterone receptor (PR), make up about 70% of all breast cancers [1]. Advances in the understanding of the biological heterogeneity of breast cancer have driven the development of novel therapeutic strategies, resulting in substantial improvements in patient survival. Despite all these developments, advanced breast cancer continues to be a major cause of cancer‐related death among women worldwide [2, 3]. Therefore, finding biomarkers to identify patients who are more prone to progress to advanced disease and would be better served with more aggressive treatments is critical.

The analysis of genes important in the invasion and metastasis pathways in hormone receptor‐positive breast tumors can help identify patients more likely to progress to advanced disease and develop prognostic biomarkers. In routine clinical practice, ER, PR (which are markers of ER signaling pathway), HER2 (which is a marker of HER2 signaling pathway), and ki67 (which is a marker of cancer cell proliferation pathway) are analyzed for every breast cancer patient by immunohistochemistry (IHC) [4]. Still, no marker of cellular invasion and metastasis is routinely checked [5]. The analysis of gene expression has been used to classify breast tumors into clinically useful risk groups [6, 7]. Several commercial breast cancer gene expression signatures have been developed, including the 21‐gene signature (Oncotype DX) [8], the 70‐gene signature (MammaPrint) [9], and the 50‐gene signature (PAM50) [10].

Because of the high costs, these tests are unavailable in most low‐ to middle‐income countries. In order to develop an in‐house test, we selected five genes (*MMP11*, *MYBL2, SCUBE2, CTSV*, and *CD68*) that are involved in tumor invasion and metastasis. These genes are incorporated in multiple breast cancer gene expression profiles: *MMP11* (Oncotype DX, PAM50), *MYBL2* (Oncotype DX, PAM50), *SCUBE2* (Oncotype DX, MammaPrint), *CTSV*, and *CD68* (Oncotype DX). All of these genes are included in the Oncotype DX test, which examines the expression of 16 genes. This test is validated to predict the likelihood of chemotherapy benefit in early‐stage, hormone receptor‐positive breast cancer [8, 11, 12]. The five‐gene assay, compared to commercial gene signatures, has reduced costs, a more simplified design, and potential for local adaptation.

Matrix metalloproteinases (MMPs) are zinc‐dependent endoproteases that can degrade extracellular matrix (ECM) proteins. MMPs are the most significant protease family involved in tumorigenesis, invasion, and metastasis [13, 14]. MMP11 is anchored to the cell membrane, and its expression in invasive breast tumors is higher than in normal tissue [15]. MMP11 expression is restricted to the stromal cells surrounding invasive breast cancer cells, but not the in situ component of the breast carcinomas [16]. Cysteine proteinases (cathepsins) are proteolytic enzymes within the lysosomes that are important in extracellular matrix degradation during tumor invasion and metastasis [17]. *CTSV* (that encodes cathepsin V, also known as cathepsin L2), is overexpressed in breast carcinomas [18], and its elevated expression is associated with adverse prognosis. *CTSV* is expressed in both breast cancer cells and the surrounding stroma [19].

Tumor‐associated macrophages (TAMs) regulate tumor cells and their microenvironment, and are important in controlling tumor growth and response to therapy [20, 21, 22, 23]. CD68 is considered a marker of TAM, and its expression is associated with reduced survival in hormone receptor‐positive breast cancers [24, 25]. Signal peptide, CUB domain, and EGF‐like domain‐containing 2 (SCUBE2) protein is a member of the evolutionary conserved SCUBE gene family. When overexpressed, it is a secreted glycoprotein that remains stably associated with the cell surface. *SCUBE2* overexpression in invasive breast carcinomas is associated with better prognosis through inhibition of breast cancer cell migration and invasion [26, 27]. *MYBL2* amplification is observed in 9%–13% of breast cancers and drives abnormal cell proliferation and invasion [28]. *MYBL2* overexpression disrupts cell adhesion and promotes epithelial‐mesenchymal transition (EMT), increasing the metastatic potential of breast tumors [29].

In the present study, we analyzed the expression of five genes to identify a gene expression pattern associated with well‐established pathology markers indicative of poor prognosis in early‐stage hormone receptor‐positive breast cancers. We also analyzed the amplification of *MYBL2* gene by Fluorescence In Situ Hybridization (FISH) method.

## 2 Methods

### 2.1 Study Cohort

We screened 276 hormone receptor‐positive breast cancer patients who received treatment in several oncology clinics and hospitals in Tehran (including Tajrish Shohada Hospital, Mehrad Hospital, and Erfan Hospital), Ahvaz, Kermanshah, and Karaj from February 2022 to October 2025. The study was a case series. Inclusion criteria were female breast cancer patients over the age of 18 with a diagnosis of invasive hormone receptor‐positive early‐stage breast cancer. Early‐stage breast cancer was defined as non‐metastatic cancer with 0–3 axillary lymph node involvement. Patients were required to have FFPE (formalin‐fixed paraffin‐embedded) samples from the primary tumor. Exclusion criteria were ER‐negative, ER‐low (< 10% stained cells), HER2‐positive tumors, prior neoadjuvant chemotherapy and the absence of invasive breast carcinoma in the available FFPE tissue blocks. The institutional review board of cancer research center, Shahid Beheshti University of Medical Sciences, approved the study (IR.SBMU.CRC.REC.1403.007), and informed consent was obtained from all patients in accordance with the recommendations of our Institutional Review Board (IRB). The study specimens had previously undergone immunohistochemical (IHC) evaluation for ER, PR, HER2, and Ki67. Clinical and pathological variables analyzed included age of cancer diagnosis, tumor histology, grade, focality, size, axillary nodal status, lympho‐vascular invasion, tumor stage, ER, PR, HER2, and Ki67 IHC status. Fluorescence In Situ Hybridization (FISH) was conducted on samples that yielded a 2+ score in HER2 IHC testing.

### 2.2 Fluorescence In Situ Hybridization (FISH)

FISH for HER2 (*ERBB2*) gene was performed using HER2 dual probe kit (CytoCell, Oxford Gene Technology Inc, UK). HER2 and centromere 17 signals were analyzed using ASI HiFISH software (Applied Spectral Imaging, CA, USA). Signal quantification was performed across 40 nuclei sampled from the invasive breast carcinoma. HER2 amplification status was determined in accordance with the 2023 American Society of Clinical Oncology/College of American Pathologists (ASCO/CAP) guidelines [30].

FISH for *MYBL2* gene was performed using *MYBL2* FISH probe (Empire Genomics, New York, USA). *MYBL2* signals were analyzed using ASI HiFISH software. A total of 40 nuclei from invasive breast tumor cells were evaluated for signal enumeration. Samples with an average of ≥ 6 *MYBL2* signals per cell were considered *MYBL2* amplified, while having 3‐5 copies was defined as copy number gain.

### 2.3 RNA Extraction and Purification

A representative section from each specimen was stained with hematoxylin and eosin (H&E) to identify the invasive tumor component and assess the proportion of tumor cells within the tissue. Samples containing < 50% invasive tumor content underwent macrodissection to increase purity. Total RNA was subsequently isolated from three 10‐µm formalin‐fixed paraffin‐embedded (FFPE) sections using the Qiagen RNeasy FFPE kit (QIAGEN, Hilden, Germany), following the manufacturer's protocols. Briefly, tumor sections underwent dewaxing in xylene (Merck Millipore, Germany). RNA release was initiated via incubation at 56°C in a lysis buffer supplemented with proteinase K, followed by an 80°C incubation step to reverse formalin‐induced crosslinking. Genomic DNA was removed through DNase treatment. The resulting lysate was processed using an RNeasy MinElute spin column, where RNA was captured on the membrane, washed of contaminants, and finally eluted in RNase‐free water. RNA concentration and purity were assessed using a Qubit RNA High Sensitivity kit (Thermo Fisher Scientific, CA, USA) and a NanoDrop spectrophotometer (Thermo Fisher Scientific, OR, USA), respectively. To ensure the absence of residual DNA, the TaqMan ACTB assay was utilized, incorporating genomic DNA from breast tissue as a positive control and a no‐template sample as a negative control. Any samples exhibiting DNA contamination underwent secondary treatment with DNase I (DNA‐free kit, Thermo Fisher Scientific, OR, USA) before re‐assessment.

### 2.4 Reverse Transcription and Real‐Time PCR

Complementary DNA (cDNA) was synthesized from the extracted RNA using the Omniscript RT kit and random hexamers (Qiagen, Hilden, Germany), with reaction mixtures incubated at 37°C for 60 min. TaqMan gene expression assays (Thermo Fisher Scientific, Pleasanton, CA) were used for expression analysis of *MMP11*, *CTSV*, *CD68*, *SCUBE2*, and *MYBL2*, with normalization performed using five established reference genes for breast tissue: *ACTB*, *GAPDH*, *GUSB*, *RPLP0*, and *TFRC* [31]. (Supporting Table 1).

Triplicate quantitative real‐time PCR (qPCR) reactions were carried out using the QuantStudio 3 system (Thermo Fisher Scientific, CA, USA). Each 20 µL amplification reaction utilized the qPCRBIO Probe Mix Lo‐ROX (PCR Biosystems Ltd., London, UK). The thermal cycling profile consisted of an initial denaturation at 95°C for 2 min, followed by 40 cycles of 95°C for 5 s and 60°C for 25 s. The ΔCt (cycle threshold) method was used to calculate gene expression, with ΔCt defined as the difference between the Ct values of the reference genes and the target gene (Ct_reference − Ct_target). Normalization was based on the mean Ct of the five reference genes using QuantStudio 3 software.

### 2.5 Statistical Methods

Descriptive statistical analyses were performed to characterize the study population, and summarize baseline demographic and clinical variables. The normality of continuous variables was assessed using the Kolmogorov–Smirnov test to guide the selection of appropriate parametric or non‐parametric statistical methods. All statistical tests were conducted using two‐sided hypotheses. A *p*‐value of < 0.05 was considered statistically significant, indicating a meaningful association between mRNA expression and clinicopathological factors.

The following clinicopathological characteristics were dichotomized: age at cancer diagnosis ≤ 50 versus > 50 (to assess its impact on mRNA expression), tumor size (to evaluate its relationship with expression levels), histologic grade (to determine if expression varies by tumor differentiation), lymph node status (to examine its prognostic significance), and histologic subtype (to compare between different breast cancer types).

The relationship between mRNA expression levels and clinicopathological variables was evaluated using the independent‐samples *t*‐test for normally distributed data to compare mean expression levels between groups and the Wilcoxon rank‐sum test for non‐normally distributed data to evaluate median differences between groups. Bonferroni correction was used to compensate for multiple comparisons.

Statistical analysis was performed using SPSS version 27.0.1 (IBM Corp., Armonk, NY) and R Statistical Software version 4.3.1 (Vienna, Austria).

### 2.6 Machine Learning Analysis

To identify the optimal predictive model for nodal involvement, 12 supervised machine learning algorithms were developed and compared, including Random Forest, XGBoost, Logistic Regression, SGD Classifier, Decision Tree, Extra Trees, Gradient Boosting, AdaBoost, LightGBM, Multi‐Layer Perceptron (MLP), k‐Nearest Neighbors (kNN), and Naive Bayes classifiers. All models were implemented using Python (scikit‐learn and imbalanced‐learn). The dataset was randomly split into training (80%) and test (20%) sets, maintaining class balance through stratified sampling. Because the number of node‐positive cases was smaller than the number of node‐negative cases, the SMOTEENN hybrid resampling technique was applied to the training data. SMOTEENN combines Synthetic Minority Oversampling (SMOTE) with Edited Nearest Neighbor (ENN) undersampling to both oversample the minority class and remove ambiguous samples near decision boundaries, enhancing sensitivity and generalization. Each model was trained using a pipeline integrating the resampling step and classifier. Hyperparameters were optimized using GridSearchCV with five‐fold stratified cross‐validation, with the F_2_‐score as the optimization metric (a metric that weights recall (sensitivity) higher than precision to prioritize identification of node‐positive patients). The F_2_‐score was defined as:

$$F_{\beta }=\frac{\left(1+\beta^{2})\times (\text{Precision}\times \text{Recall}\right)}{(\beta^{2}\times \text{Precision})+\text{Recall}},\beta =2$$

This metric was used as the primary optimization criterion. For each model, the best‐performing hyperparameter combination was identified, and the final model was evaluated on the held‐out test set. Performance metrics included sensitivity (recall), specificity, F_2_‐score, F_1_‐score, and ROC‐AUC. A confusion matrix was also generated for each model to visualize classification outcomes (true positives, false positives, true negatives, and false negatives).

## 3 Results

### 3.1 Patient Characteristics

Out of 276 initially identified patients, 242 met the study selection criteria, with 239 ultimately included in the final analysis. Two patients were excluded after initial selection: one due to the presence of ductal carcinoma in situ (DCIS) only without invasive breast tumor in the FFPE block, and one due to insufficient RNA yield (Supporting Figure 1). One sample was ER‐low‐positive; therefore, it was excluded from the study. All tumor samples were obtained from surgical resections, and the corresponding FFPE blocks had been stored for 1 to 6 months prior to analysis. Patient characteristics are summarized in Table 1. Patients had a median age of 50 years at breast cancer diagnosis (IQR, 43–60 years). Breast cancer was unilateral and unifocal in 219 patients (91.6%). Two hundred and fourteen (89.5%) patients presented with invasive ductal carcinoma, and seventeen (7.1%) presented with invasive lobular carcinoma. 230 tumors (96.2%) were PR‐positive, 8 tumors (3.3%) were PR‐low‐positive, and 9 tumors (3.8%) were PR negative.

**Table 1:** Clinical and Pathological characteristics of the patients (*N* = 239).

Variable	No.	%
**Age at breast cancer diagnosis**		
Median (IQR)	50 (43–60)	
≤ 50	126	52.7
> 50	113	47.3
**Tumor histology**		
IDC	214	89.5
ILC	17	7.1
Mucinous	5	2.1
Other*	3	1.3
**Tumor focality**		
Unifocal	219	91.6
Multifocal	17	7.1
Bilateral	3	1.3
**Tumor grade**		
1	53	22.2
2	169	70.7
3	14	5.9
Missing	3	1.3
**Tumor size (cm)**		
≤ 1	39	16.3
> 1–2	91	38.1
> 2–4	102	42.7
> 4	4	1.7
Missing	3	1.3
**Nodal status**		
0	216	90.4
1–3	23	9.6
**Lympho‐vascular Invasion**		
Present	39	16.3
Absent	197	82.4
Missing	3	1.3
**Tumor infiltrating lymphocytes (TIL)**		
< 20%	104	43.5
20–50%	25	10.5
> 50%	1	0.4
Missing	109	45.6
**Stage**		
I	121	50.6
II	113	47.3
III	2	0.8
Missing	3	1.3
**ER IHC Nuclei Staining**		
10–50%	8	3.3
> 50%	219	91.6
ER staining percentage missing (reported as ER positive)	12	5
**PR IHC Nuclei Staining**		
< 1%	9	3.8
1–10%	8	3.3
11–50%	58	24.3
> 50%	153	64
PR staining percentage Missing (reported as PR positive)	11	4.6
**HER2 IHC Score**		
0	137	57.3
1	61	25.5
2	39	16.3
3	0	0
HER2 score missing (reported as HER2 Negative)	2	0.8
**Ki67 IHC Staining**		
≤ 5%	20	8.4
6–29%	170	71.1
≥ 30%	42	17.6
Missing	7	2.9

Abbreviations: ER, Estrogen receptor; FISH, Fluorescence In Situ Hybridization; HER2, Human epidermal growth factor receptor 2; IDC, Invasive ductal carcinoma; IHC, Immunohistochemistry; ILC, Invasive lobular carcinoma; IQR, Interquartile range; PR, Progesterone receptor.

*Invasive apocrine cancer, Invasive micropapillary cancer, Invasive tubular cancer.

### 3.2 FISH Analysis

**Figure 1:**
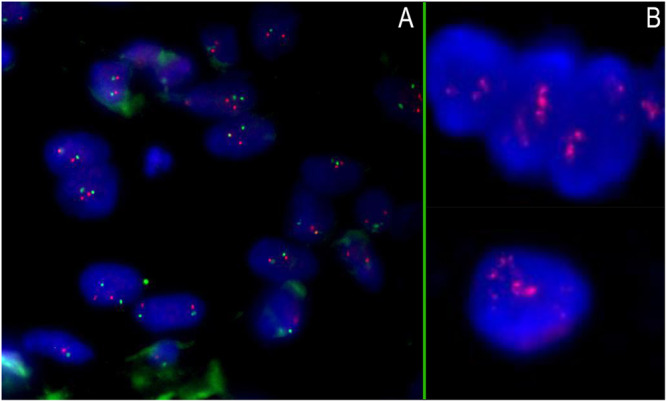
(A) Illustration of two‐color FISH (fluorescent in situ hybridization) analysis of HER2, representing centromere 17 (green) and HER2 (*ERBB2*) gene (red) of a sample without amplification, and (B) FISH analysis of *MYBL2* gene of a tumor exhibiting *MYBL2* copy number gain.

HER2 FISH was performed for 40 samples with a 2 + HER2 IHC. One specimen demonstrated HER2 amplification by FISH and was therefore excluded from the analysis. A representative case with a normal HER2 copy number is shown in Figure 1A.

*MYBL2* FISH was performed for all samples. We identified one sample with *MYBL2* amplification, and four cases with *MYBL2* copy number gain. A representative case with *MYBL2* copy number gain is depicted in Figure 1B.

### 3.3 Differential Expression of the Selected Genes in Tumor Samples

**Figure 2:**
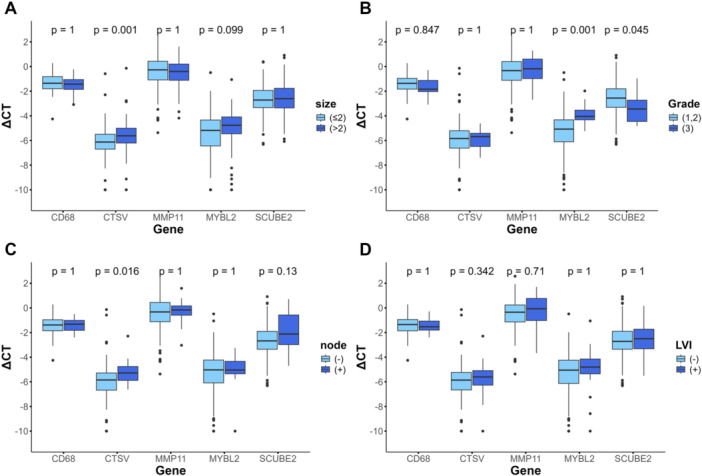
Boxplot of the association between gene expression and tumor pathologic characteristics. (A) mRNA ΔCt values/tumor size > 2 cm or ≤ 2 cm; (B) mRNA ΔCt values/tumor grade; (C) mRNA ΔCt values/Lymph node involvement; (D) mRNA ΔCt values/lympho‐vascular invasion. Ct, Cycle threshold; LVI, Lympho‐vascular invasion.

**Figure 3:**
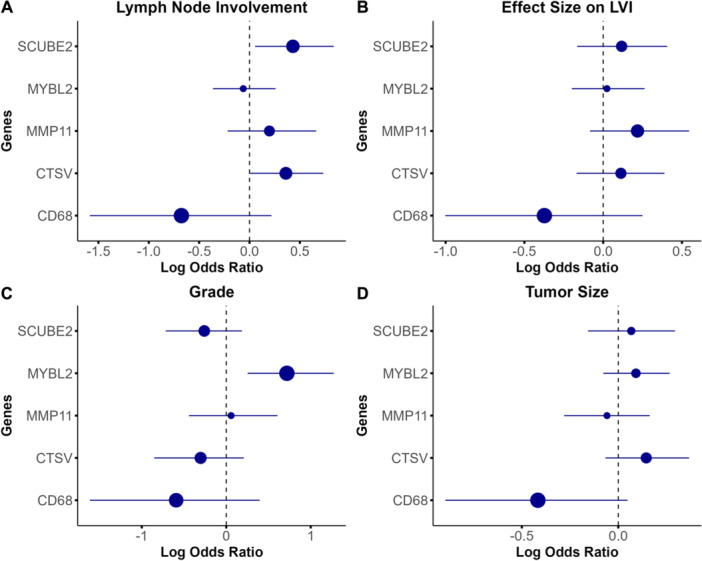
Multivariable analysis of the association of the expression of all five genes (*CTSV*, *MMP11*, *MYBL2*, *SCUBE2*, and CD68) and (A) Lymph node involvement, (B) lympho‐vascular invasion, (C) tumor grade, and (D) tumor size. LVI, Lympho‐vascular invasion

**Figure 4:**
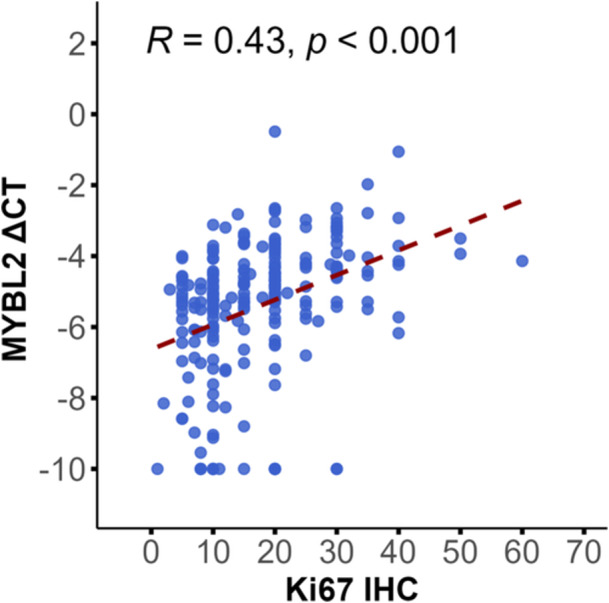
Scatter plots of *MYBL2* mRNA ΔCt values and ki67 IHC. Ct, Cycle threshold; IHC, Immunohistochemistry.

The association of expression levels of the selected genes and pathological characteristics is presented in Figure 2. Expression levels of *CTSV* gene were significantly associated with increasing tumor size (*p* = 0.001) and lymph node involvement (*p* = 0.016). Expression levels of *MYBL2* gene were associated with higher tumor grade (*p* = 0.001). Expression levels of *SCUBE2* gene were inversely associated with tumor grade (*p* = 0.04). Higher expression of *CTSV* and *MMP11* was associated with a trend toward a greater frequency of lympho‐vascular invasion, although the association was not statistically significant. Multivariable logistic regression analysis of all five genes and age demonstrated that lymph node involvement was significantly associated with *CTSV* gene expression (*p* = 0.049) and age (*p* < 0.001) (Figure 3). The percentage of Ki67‐positive tumor cells was moderately correlated with MYBL2 mRNA expression (r = 0.43, *p* < 0.001). (Figure 4, Supporting Figure 2). Table 2 summarizes the gene functions and their clinicopathological associations.

**Table 2:** The function and clinical association of the selected genes (Univariate analysis).

Gene	Function	Association with pathological markers
*CTSV*	Extracellular matrix degradation during tumor invasion and metastasis	Increased tumor size, More lymph node involvement
*MMP11*	Extracellular matrix degradation during tumor invasion and metastasis	None
*MYBL2*	Abnormal cell proliferation and invasion, cell adhesion disruption and epithelial‐mesenchymal transition promotion	Increased tumor grade
*SCUBE2*	Inhibition of tumor cell migration and invasion	Decreased tumor grade
*CD68*	CD68 is considered a marker of TAMs, which regulate tumor cells and their microenvironment	None

Abbreviation: TAMs, Tumor‐associated macrophages.

A post‐hoc power analysis was performed to determine the statistical power for detecting the observed difference in *CTSV* gene expression between lymph node‐negative and lymph‐node‐positive patients. Based on the observed mean ∆Ct difference of 0.847, the respective standard deviations (1.503 and 0.966), and a significance level (α) of 0.05, the achieved power of the study was 96.3%.

### 3.4 Receiver‑Operator Characteristic Analyses to Predict Lymph Node Involvement

**Figure 5:**
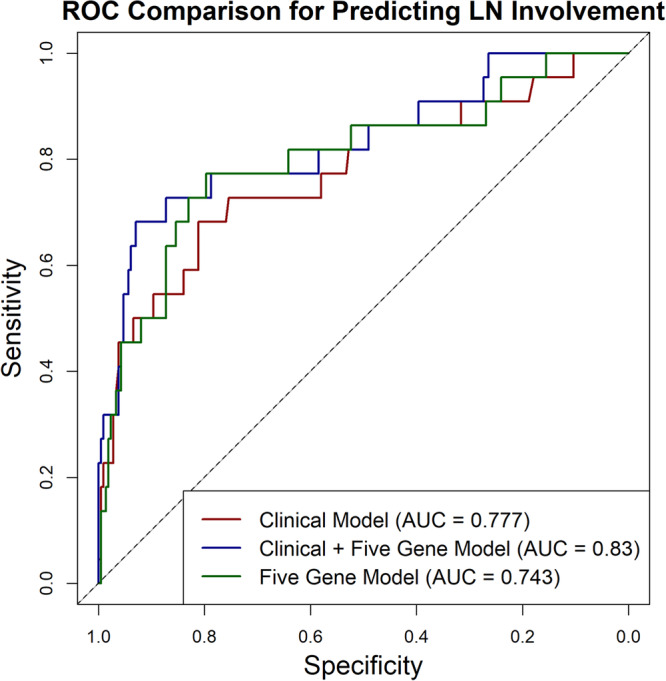
Receiver‐operator characteristic (ROC) analysis. **Red Line.** ROC analysis for predicting lymph node involvement using standard clinicopathologic features (age at cancer diagnosis, tumor size, and histologic grade), AUC = 0.777. **Green Line.** ROC analysis for predicting lymph node involvement using the five gene model, AUC = 0.743. **Blue Line.** ROC analysis for predicting lymph node involvement, clinicopathological model with the five‐gene model, AUC = 0.83.

Receiver‐operator characteristic (ROC) analysis was used to predict lymph node involvement using standard clinicopathologic features (age at cancer diagnosis, tumor size, and histologic grade), and five gene expression markers (*CD68*, *CTSV*, *MMP11*, *MYBL2*, and *SCUBE2*). ROC analysis to predict lymph node involvement using the five gene expression model resulted in AUC = 0.743. ROC analysis to predict lymph node involvement using the clinical model resulted in AUC = 0.777. Integrating the clinicopathological model with the five‐gene model increased the AUC to 0.83 (Figure 5).

### 3.5 Evaluation of Machine Learning Models in Predicting Nodal Status

**Figure 6:**
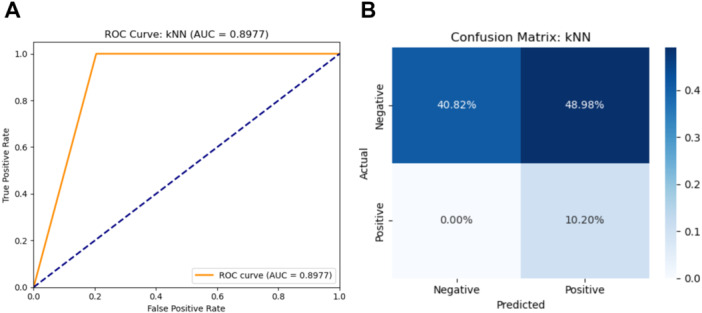
Machine learning analysis for predicting lymph node involvement. (A) Receiver‐operator characteristic (ROC) analysis, (B) Confusion matrix.

Among the 12 machine learning models trained on 190 samples, the k‐nearest neighbors (kNN) model achieved the best performance, with an area under the ROC curve (AUC) of 0.90. This model was developed to predict lymph node involvement in hormone receptor–positive early breast cancer using three clinicopathologic features (age at cancer diagnosis, tumor size, and histologic grade) and five gene expression markers (*CD68*, *CTSV*, *MMP11*, *MYBL2*, and *SCUBE2*). The kNN model demonstrated excellent sensitivity (100%), but moderate specificity (45.5%) on the test set with 49 patients. The ROC‐AUC score was 0.898, indicating strong overall discriminative ability. The confusion matrix and ROC curve are presented in Figure 6.

## 4 Discussion

Univariate analysis of the present study samples demonstrated a significant association between expression levels of *CTSV* (encoding cathepsin V), *MYBL2* (encoding myb‐related protein B), and *SCUBE2* (encoding signal peptide, CUB and EGF‐like domain‐containing protein 2) mRNAs and the clinicopathological characteristics of early‐stage hormone receptor‐positive breast tumors. *CTSV* exhibited significantly higher expression levels in breast tumors with larger size and lymph node involvement, and there was a trend toward more lympho‐vascular invasion. Toss et al. analyzed breast ductal carcinoma in situ (DCIS) specimens with immunohistochemistry for cathepsin V protein. Cathepsin V expression was associated with larger DCIS size. DCIS associated with invasive breast cancer showed higher cathepsin V expression compared to DCIS alone. They showed that higher expression of cathepsin V is associated with poor prognosis, which is similar to our findings regarding *CTSV* mRNA expression associated with poor prognostic pathology markers [22]. The interaction between cancer cells and their surrounding stroma is among the hallmarks of cancer [32]. Cathepsin V exhibits extensive elastase activity and is expressed in human activated macrophages [33], although its expression in cancerous tissue macrophages has not been documented in breast cancer. Toss et al. showed that cathepsin V is expressed in the cytoplasm of breast cancer cells and cancer‐associated fibroblasts (CAFs) [19]. Sun et al. analyzed the expression of different cathepsins in breast tumors and reported that cathepsin V expression is associated with distant metastasis [34]. Sereesongsaeng et al. showed that cathepsin V silencing by shRNA significantly reduced tumor cell invasion in hormone receptor‐positive breast cancer cell lines [35]. Cathepsin V directly affects the basement membrane and extracellular matrix degradation, facilitating tumor cell invasion into the surrounding stroma. Our findings highlight the interplay between tumor cells and the surrounding stromal microenvironment during tumor progression, in which *CTSV* gene (which encodes cathepsin V) may have a significant role. Multivariable analysis of the five gene expression model showed that *CTSV* expression analysis can predict axillary lymph node involvement.

Matrix metalloproteinases (MMPs) are a family of zinc‐dependent endopeptidases capable of degrading components of the extracellular matrix to promote invasion and metastasis of tumor cells [36]. This study did not identify a significant association between lymph node involvement and *MMP11* expression. Previous studies have shown that overexpression of MMP11 is associated with poor survival [37]. Matrix metalloproteinase 11 is expressed in stromal cells of invasive breast tumors. While most MMPs are secreted as inactive enzymes, MMP11 is processed in the cytoplasm and secreted as active, making it important in tumor invasion and metastasis [38]. Cheng et al. showed that overexpression of MMP11 is associated with poorly differentiated breast cancers and lymph node metastasis [15]. We did not find a significant association between *MMP11* gene expression, tumor grade, and lymph node involvement. This may be due to the small number of lymph node‐positive tumors in our cohort. In a more recent study, Cheng et al. demonstrated that MMP11 expression is associated with CD8 + T cell and macrophage infiltration within the breast tumor [39]. In contrast, Kim et al. reported low tumor‐infiltrating lymphocytes and worse survival in breast cancers with high MMP11 expression [36]. We did not find a significant association between tumor‐infiltrating lymphocytes and *MMP11* expression.

In the present study, expression of *MYBL2* was concordant with ki67 IHC percentage and was associated with higher tumor grade. MYBL2 is a transcription factor important in tumor proliferation and progression [40]. *MYBL2* is frequently amplified in breast tumors, and previous studies have shown that its overexpression is associated with poor prognosis [29, 41]. Although there is no clear definition for *MYBL2* gene amplification in breast cancer, based on previously published studies [42, 43, 44], we defined *MYBL2* amplification as the presence of ≥ 6 copies per cell and *MYBL2* copy number gain as the presence of 3‐5 copies per cell. In this patient cohort, we identified *MYBL2* amplification in only one sample, and three samples showed *MYBL2* copy number gain. Ginestier et al. analyzed the chromosomal locus 20q13 (which includes *MYBL2* gene) in 466 breast cancer samples and detected *MYBL2* amplification in 4% of samples [45]. The detection of *MYBL2* amplification in only a single sample in our cohort is likely attributable to tumor subtype distribution. As reported by Li et al., *MYBL2* is predominantly overexpressed in non‐luminal breast cancers, particularly the basal‐like subtype, whereas its expression is typically low in luminal tumors. Given that our cohort is mainly composed of luminal cases, the low frequency of *MYBL2* amplification is therefore expected [46]. The tumors included in this study cohort are all luminal breast cancers, and *MYBL2* amplification may not be a common mechanism in luminal tumors. In addition, there is no standard definition for *MYBL2* amplification. All four samples reported in this study, as *MYBL2* copy number gain showed high copy number gain. Other studies may have different definitions of *MYBL2* amplification.

We found an association between lower expression of *SCUBE2* gene and higher tumor grade. Cheng et al. analyzed the expression of SCUBE2 protein in breast cancer patients and found an association with a favorable outcome [26]. Overexpression of SCUBE2 protein inhibits breast tumor cell migration and invasion by increasing epithelial E‐cadherin‐containing adherence junction and reversal of epithelial‐mesenchymal transition (EMT) [27]. Association of decreased expression of *SCUBE2* gene and poor prognosis has been previously shown in other cancers, including gastric, colorectal, and brain cancers [47, 48, 49].

In the present study, we used a five‐gene model to develop a lightweight, efficient diagnostic approach using minimal materials to predict lymph node involvement in early‐stage hormone receptor‐positive breast cancer. We acknowledge the value of highly multiplexed assays in identifying prognostic/predictive biomarkers, especially when combined with machine learning and AI [50, 51]. This five‐gene panel includes genes that are incorporated in the established breast cancer gene expression assays. One gene is included in MammaPrint, two genes in PAM50, and all five genes are included in the Oncotype DX test. All of these assays have prognostic value in early‐stage hormone receptor‐positive breast cancer. In addition, the Oncotype DX test can predict the benefit of adding chemotherapy to hormonal therapy in this group of patients.

Our study has limitations. The study is cross‐sectional, assessing gene expression and pathological markers at a single time point. It does not provide information on long‐term patient outcomes (e.g., recurrence, metastasis, survival), which limits its predictive clinical usefulness. All analyses are correlative; the study does not include functional experiments (e.g., knockdown/overexpression studies, pathway analysis) to elucidate causal mechanisms behind gene expression changes and tumor aggressiveness. In addition, our study does not delve deep into the full spectrum of the tumor microenvironment (such as stromal or immune cell phenotyping). Other limitations of this study include the small number of genes compared to validated signatures and the exploratory nature of the correlations with pathology markers. In addition, independent external validation has not yet been performed.

In conclusion, we found a significant association between *CTSV*, *MYBL2*, and *SCUBE2* gene expression and clinicopathological features. Our study reveals that analysis of the expression of *CTSV* gene can help identify early‐stage hormone receptor‐positive breast cancers that are more likely to metastasize; therefore, they need more intense treatment modalities. In addition, we found that higher *MYBL2* and lower *SCUBE2* gene expression are associated with more aggressive breast tumors. Integrating the clinicopathological model with the five‐gene model increased the ability of each model to predict lymph node involvement. Further studies are needed to analyze the association of this five‐gene model with survival outcomes, such as overall survival and progression‐free survival. If validated in independent cohorts, it can be clinically implemented as an in‐house low‐cost alternative to commercial expression profiling tests.

## Author Contributions

**Shayan Forghani:** methodology, data curation, investigation, formal analysis, writing – original draft. **Hamid Rezvani:** conceptualization, supervision, project administration, writing – review and editing. **Arman Forghani:** methodology, data curation, investigation, formal analysis. **Fatemeh Mahdavi Sabet:** methodology, data curation, investigation, formal analysis. **Atieh Akbari:** conceptualization, supervision, writing – review and editing. **Sanaz Tabarestani:** conceptualization, methodology, investigation, supervision. **Hamid Reza Mirzaee:** conceptualization, writing – review and editing, supervision.

## Funding

The authors have nothing to report.

## Ethics Statement

The study was approved by the ethics committee of cancer research center (IR.SBMU.CRC.REC.1403.007).

## Conflicts of Interest

The authors declare no conflicts of interest.

## Supporting information


**Figure S1:** Patient selection diagram. This diagram provides an outline of the patients selected and those excluded due to the lack of consent, tumor FFPE block unavailable, no invasive tumor in the available FFPE block, ER‐low positive tumors, or RNA extraction not successful. FFPE: Formalin‐Fixed Paraffin‐Embedded; DCIS: Ductal carcinoma in situ.

**Figure S2:** Scatter plots of mRNA ΔCt values and ki67 IHC. **A)** *CTSV* mRNA; **B)** *CD68* mRNA. **C)** *SCUBE2* mRNA. **D)** *MMP11* mRNA. Ct: Cycle threshold; IHC: Immunohistochemistry.

**Supporting File:** hsr272780‐sup‐0003‐Supplementary_Table_1.docx.

## Data Availability

The data that support the findings of this study are available from the corresponding author upon reasonable request. The datasets generated and/or analyzed during the current study are available from the corresponding author upon reasonable request.

## References

[1] N. P. McAndrew and R. S. Finn , “Clinical Review on the Management of Hormone Receptor‐Positive Metastatic Breast Cancer,” JCO Oncology Practice 18, no. 5 (2022): 319–327. 34637323 10.1200/OP.21.00384

[2] R. L. Siegel , K. D. Miller , N. S. Wagle , and A. Jemal , “Cancer Statistics, 2023,” CA: A Cancer Journal for Clinicians 73, no. 1 (2023): 17–48. 36633525 10.3322/caac.21763

[3] S. Forghani , H. R. Mirzaee , H. Rezvani , et al., “The Patterns and Spectrum of BRCA1 and BRCA2 Mutations in Iranian Breast and Ovarian Cancer Patients,” Familial Cancer 24, no. 2 (2025): 34. 40159529 10.1007/s10689-025-00459-7

[4] F. Mahdavi Sabet , F. Zeinalkhani , S. Forghani , P. Kamali Hakim , H. Zeinalkhani , and F. Tahanian , “Associations Between Apparent Diffusion Coefficient Values and Histopathologic Prognostic Factors in Breast Cancer: A Retrospective Study,” International Journal of Breast Cancer 2026, no. 1 (2026): 5114852. 42094985 10.1155/ijbc/5114852 PMC13141677

[5] H. Rezvani , S. Forghani , A. Forghani , F. M. Sabet , A. Akbari , and S. Tabarestani , “Assessment of ESR1, PGR, ERBB2, and MKI67 mRNA in Hormone Receptor‐Positive Early Breast Cancer: A Cross‐Sectional Study,” Health Science Reports 8, no. 7 (2025): e71062. 40666735 10.1002/hsr2.71062 PMC12261032

[6] S. Tabarestani , S. M. H. Ghaderian , H. Rezvani , and R. Mirfakhraie , “Expression Profiling of Breast Cancer Patients Treated With Tamoxifen: Prognostic or Predictive Significance,” Medical Oncology 31, no. 4 (2014): 896. 24563328 10.1007/s12032-014-0896-5

[7] H. Rezvani , S. Forghani , A. Forghani , F. M. Sabet , A. Akbari , and S. Tabarestani , “Assessment of ESR1, PGR, ERBB2, and MKI67 mRNA in Hormone Receptor‐Positive Early Breast Cancer: A Cross‐Sectional Study,” Health Science Reports 8, no. 7 (2025): e71062. 40666735 10.1002/hsr2.71062 PMC12261032

[8] S. Paik , S. Shak , G. Tang , et al., “A Multigene Assay to Predict Recurrence of Tamoxifen‐Treated, Node‐Negative Breast Cancer,” New England Journal of Medicine 351, no. 27 (2004): 2817–2826. 15591335 10.1056/NEJMoa041588

[9] L. J. van 't Veer , H. Dai , M. J. van de Vijver , et al., “Gene Expression Profiling Predicts Clinical Outcome of Breast Cancer,” Nature 415, no. 6871 (2002): 530–536. 11823860 10.1038/415530a

[10] T. Sørlie , R. Tibshirani , J. Parker , et al., “Repeated Observation of Breast Tumor Subtypes in Independent Gene Expression Data Sets,” Proceedings of the National Academy of Sciences 100, no. 14 (2003): 8418–8423. 10.1073/pnas.0932692100 PMC166244 12829800

[11] J. A. Sparano , R. J. Gray , D. F. Makower , et al., “Prospective Validation of a 21‐Gene Expression Assay in Breast Cancer,” New England Journal of Medicine 373, no. 21 (2015): 2005–2014. 26412349 10.1056/NEJMoa1510764 PMC4701034

[12] J. A. Sparano , R. J. Gray , D. F. Makower , et al., “Adjuvant Chemotherapy Guided by a 21‐Gene Expression Assay in Breast Cancer,” New England Journal of Medicine 379, no. 2 (2018): 111–121. 29860917 10.1056/NEJMoa1804710 PMC6172658

[13] K. Kessenbrock , V. Plaks , and Z. Werb , “Matrix Metalloproteinases: Regulators of the Tumor Microenvironment,” Cell 141, no. 1 (2010): 52–67. 20371345 10.1016/j.cell.2010.03.015 PMC2862057

[14] M. Endres , S. Kneitz , M. F. Orth , R. K. Perera , A. Zernecke , and E. Butt , “Regulation of Matrix Metalloproteinases (MMPs) Expression and Secretion in MDA‐MB‐231 Breast Cancer Cells by LIM and SH3 Protein 1 (LASP1),” Oncotarget 7, no. 39 (2016): 64244–64259. 27588391 10.18632/oncotarget.11720 PMC5325439

[15] C. W. Cheng , J. C. Yu , H. W. Wang , et al., “The Clinical Implications of MMP‐11 and CK‐20 Expression in Human Breast Cancer,” Clinica Chimica Acta 411, no. 3–4 (2010): 234–241. 10.1016/j.cca.2009.11.009 19914229

[16] P. Basset , J. P. Bellocq , C. Wolf , et al., “A Novel Metalloproteinase Gene Specifically Expressed in Stromal Cells of Breast Carcinomas,” Nature 348, no. 6303 (1990): 699–704. 1701851 10.1038/348699a0

[17] Y. Yasuda , Z. Li , D. Greenbaum , M. Bogyo , E. Weber , and D. Brömme , “Cathepsin V, a Novel and Potent Elastolytic Activity Expressed in Activated Macrophages,” Journal of Biological Chemistry 279, no. 35 (2004): 36761–36770. 15192101 10.1074/jbc.M403986200

[18] I. Santamaría , G. Velasco , M. Cazorla , A. Fueyo , E. Campo , and C. López‐Otín , “Cathepsin L2, a Novel Human Cysteine Proteinase Produced by Breast and Colorectal Carcinomas,” Cancer Research 58, no. 8 (1998): 1624–1630. 9563472

[19] M. Toss , I. Miligy , K. Gorringe , et al., “Prognostic Significance of Cathepsin V (CTSV/CTSL2) in Breast Ductal Carcinoma In Situ,” Journal of Clinical Pathology 73, no. 2 (2020): 76–82. 31444238 10.1136/jclinpath-2019-205939

[20] R. A. Franklin and M. O. Li , “Ontogeny of Tumor‐Associated Macrophages and Its Implication in Cancer Regulation,” Trends in Cancer 2, no. 1 (2016): 20–34. 26949745 10.1016/j.trecan.2015.11.004 PMC4772875

[21] A. Mantovani and P. Allavena , “The Interaction of Anticancer Therapies With Tumor‐Associated Macrophages,” Journal of Experimental Medicine 212, no. 4 (2015): 435–445. 25753580 10.1084/jem.20150295 PMC4387285

[22] S. Tabarestani , M. E. Akbari , and S. Namaki , “Novel Approaches to Immunotherapy in Triple Negative Breast Cancer,” International Journal of Cancer Management 11 (2018): e87024.

[23] S. Tabarestani , M. E. Akbari , and S. Namaki , “Genomics Role in Cancer Immunosurveillance: Impact on Immunotherapy Response,” International Journal of Cancer Management 11, (2018): e85552.

[24] V. Pelekanou , F. Villarroel‐Espindola , K. A. Schalper , L. Pusztai , and D. L. Rimm , “CD68, CD163, and Matrix Metalloproteinase 9 (MMP‐9) Co‐Localization in Breast Tumor Microenvironment Predicts Survival Differently in ER‐Positive and ‐Negative Cancers,” Breast Cancer Research 20, no. 1 (2018): 154. 30558648 10.1186/s13058-018-1076-x PMC6298021

[25] Y. Chen , T. A. Klingen , H. Aas , E. Wik , and L. A. Akslen , “CD47 and CD68 Expression in Breast Cancer Is Associated With Tumor‐Infiltrating Lymphocytes, Blood Vessel Invasion, Detection Mode, and Prognosis,” Journal of Pathology: Clinical Research 9, no. 3 (2023): 151–164. 36598153 10.1002/cjp2.309 PMC10073931

[26] C. J. Cheng , Y. C. Lin , M. T. Tsai , et al., “SCUBE2 Suppresses Breast Tumor Cell Proliferation and Confers a Favorable Prognosis in Invasive Breast Cancer,” Cancer Research 69, no. 8 (2009): 3634–3641. 19369267 10.1158/0008-5472.CAN-08-3615

[27] Y. C. Lin , et al., “Tumor Suppressor SCUBE2 Inhibits Breast‐Cancer Cell Migration and Invasion through the Reversal of Epithelial‐Mesenchymal Transition,” Journal of Cell Science 127, no. Pt 1 (2014): 85–100. 24213532 10.1242/jcs.132779

[28] C. Ginestier , N. Cervera , P. Finetti , et al., “Prognosis and Gene Expression Profiling of 20q13‐amplified Breast Cancers,” Clinical Cancer Research 12, no. 15 (2006): 4533–4544. 16899599 10.1158/1078-0432.CCR-05-2339

[29] D. Tao , Y. Pan , G. Jiang , et al., “B‐Myb Regulates Snail Expression to Promote Epithelial‐To‐Mesenchymal Transition and Invasion of Breast Cancer Cell,” Medical Oncology 32, no. 1 (2015): 412. 25502082 10.1007/s12032-014-0412-y

[30] A. C. Wolff , M. R. Somerfield , M. Dowsett , et al., “Human Epidermal Growth Factor Receptor 2 Testing in Breast Cancer: ASCO‐College of American Pathologists Guideline Update,” Journal of Clinical Oncology 41, no. 22 (2023): 3867–3872. 37284804 10.1200/JCO.22.02864

[31] J. Vandesompele , K. De Preter , F. Pattyn , et al., “Accurate Normalization of Real‐Time Quantitative RT‐PCR Data by Geometric Averaging of Multiple Internal Control Genes,” Genome Biology 3, no. 7 (2002): research0034.1. 12184808 10.1186/gb-2002-3-7-research0034 PMC126239

[32] D. Hanahan and R. A. Weinberg , “Hallmarks of Cancer: The Next Generation,” Cell 144, no. 5 (2011): 646–674. 21376230 10.1016/j.cell.2011.02.013

[33] Y. Yasuda , Z. Li , D. Greenbaum , M. Bogyo , E. Weber , and D. Brömme , “Cathepsin V, a Novel and Potent Elastolytic Activity Expressed in Activated Macrophages,” Journal of Biological Chemistry 279, no. 35 (2004): 36761–36770. 15192101 10.1074/jbc.M403986200

[34] T. Sun , D. Jiang , L. Zhang , Q. Su , W. Mao , and C. Jiang , “Expression Profile of Cathepsins Indicates the Potential of Cathepsins B and D as Prognostic Factors in Breast Cancer Patients,” Oncology Letters 11, no. 1 (2016): 575–583. 26870250 10.3892/ol.2015.3960 PMC4727043

[35] N. Sereesongsaeng , S. H. McDowell , J. F. Burrows , C. J. Scott , and R. E. Burden , “Cathepsin V Suppresses GATA3 Protein Expression in Luminal A Breast Cancer,” Breast Cancer Research 22, no. 1 (2020): 139. 33298139 10.1186/s13058-020-01376-6 PMC7726886

[36] G. T. Brown and G. I. Murray , “Current Mechanistic Insights into the Roles of Matrix Metalloproteinases in Tumour Invasion and Metastasis,” Journal of Pathology 237, no. 3 (2015): 273–281. 26174849 10.1002/path.4586

[37] H. S. Kim , M. G. Kim , K. W. Min , U. S. Jung , and D. H. Kim , “High MMP‐11 Expression Associated With Low CD8+ T Cells Decreases the Survival Rate in Patients With Breast Cancer,” PLoS One 16, no. 5 (2021): e0252052. 34038440 10.1371/journal.pone.0252052 PMC8153507

[38] X. Zhang , S. Huang , J. Guo , et al., “Insights into the Distinct Roles of MMP‐11 in Tumor Biology and Future Therapeutics (Review),” International Journal of Oncology 48, no. 5 (2016): 1783–1793. 26892540 10.3892/ijo.2016.3400

[39] T. Cheng , P. Chen , J. Chen , Y. Deng , and C. Huang , “Landscape Analysis of Matrix Metalloproteinases Unveils Key Prognostic Markers for Patients With Breast Cancer,” Frontiers in Genetics 12 (2022): 809600. 35069702 10.3389/fgene.2021.809600 PMC8770541

[40] A. Sala , “B‐MYB, a Transcription Factor Implicated in Regulating Cell Cycle, Apoptosis and Cancer,” European Journal of Cancer 41, no. 16 (2005): 2479–2484. 16198555 10.1016/j.ejca.2005.08.004

[41] H. Shi , M. Bevier , R. Johansson , et al., “Prognostic Impact of Polymorphisms in the MYBL2 Interacting Genes in Breast Cancer,” Breast Cancer Research and Treatment 131, no. 3 (2012): 1039–1047. 22037783 10.1007/s10549-011-1826-2

[42] H. S. Yang and B. Horten , “Gain of Copy Number and Amplification of the RET Gene in Lung Cancer,” Experimental and Molecular Pathology 97, no. 3 (2014): 465–469. 25303898 10.1016/j.yexmp.2014.10.002

[43] M. Salido , L. Pijuan , L. Martínez‐Avilés , et al., “Increased ALK Gene Copy Number and Amplification Are Frequent in Non‐Small Cell Lung Cancer,” Journal of Thoracic Oncology 6, no. 1 (2011): 21–27. 21107285 10.1097/JTO.0b013e3181fb7cd6 PMC3359090

[44] K. Ohshima , K. Hatakeyama , T. Nagashima , et al., “Integrated Analysis of Gene Expression and Copy Number Identified Potential Cancer Driver Genes With Amplification‐Dependent Overexpression in 1,454 Solid Tumors,” Scientific Reports 7, no. 1 (2017): 641. 28377632 10.1038/s41598-017-00219-3 PMC5428069

[45] C. Ginestier , N. Cervera , P. Finetti , et al., “Prognosis and Gene Expression Profiling of 20q13‐amplified Breast Cancers,” Clinical Cancer Research 12, no. 15 (2006): 4533–4544. 16899599 10.1158/1078-0432.CCR-05-2339

[46] R. Li , J. Campos , and J. Iida , “A Gene Regulatory Program in Human Breast Cancer,” Genetics 201, no. 4 (2015): 1341–1348. 26510790 10.1534/genetics.115.180125 PMC4676531

[47] E. Guo , H. Liu , and X. Liu , “Overexpression of SCUBE2 Inhibits Proliferation, Migration, and Invasion in Glioma Cells,” Oncology Research Featuring Preclinical and Clinical Cancer Therapeutics 25, no. 3 (2017): 437–444. 10.3727/096504016X14747335734344 PMC7841219 27697090

[48] Q. Song , C. Li , X. Feng , et al., “Decreased Expression of SCUBE2 Is Associated With Progression and Prognosis in Colorectal Cancer,” Oncology Reports 33, no. 4 (2015): 1956–1964. 25672935 10.3892/or.2015.3790

[49] X. Wang , R. Y. Zhong , and X. J. Xiang , “Reduced Expression of SCUBE2 Predicts Poor Prognosis in Gastric Cancer Patients,” International Journal of Clinical and Experimental Pathology 11, no. 2 (2018): 972–980. 31938191 PMC6958003

[50] H. Mi , R. Varadhan , A. M. Cimino‐Mathews , L. A. Emens , C. A. Santa‐Maria , and A. S. Popel , “Spatial Architecture of Single‐Cell and Vasculature in Tumor Microenvironment Predicts Clinical Outcomes in Triple‐Negative Breast Cancer,” Modern Pathology 38, no. 2 (2025): 100652. 39522644 10.1016/j.modpat.2024.100652 PMC11845302

[51] R. Nakhli , P. A. Moghadam , and H. Mi , Sparse Multi‐Modal Graph Transformer with Shared‐Context Processing for Representation Learning of Giga‐pixel Images (IEEE/CVF Conference on Computer Vision and Pattern Recognition, 2023).

