# Supplementary figures and images for: Clinical Significance of Tumor Invasion Gene Profiling in Early‐Stage Hormone Receptor‐Positive Breast Cancer: A Cross‐Sectional Study
Source: Health Sci Rep. 2026 Jul 6;9(7):e72780. doi: 10.1002/hsr2.72780 (PMC13338577; doi:10.1002/hsr2.72780)

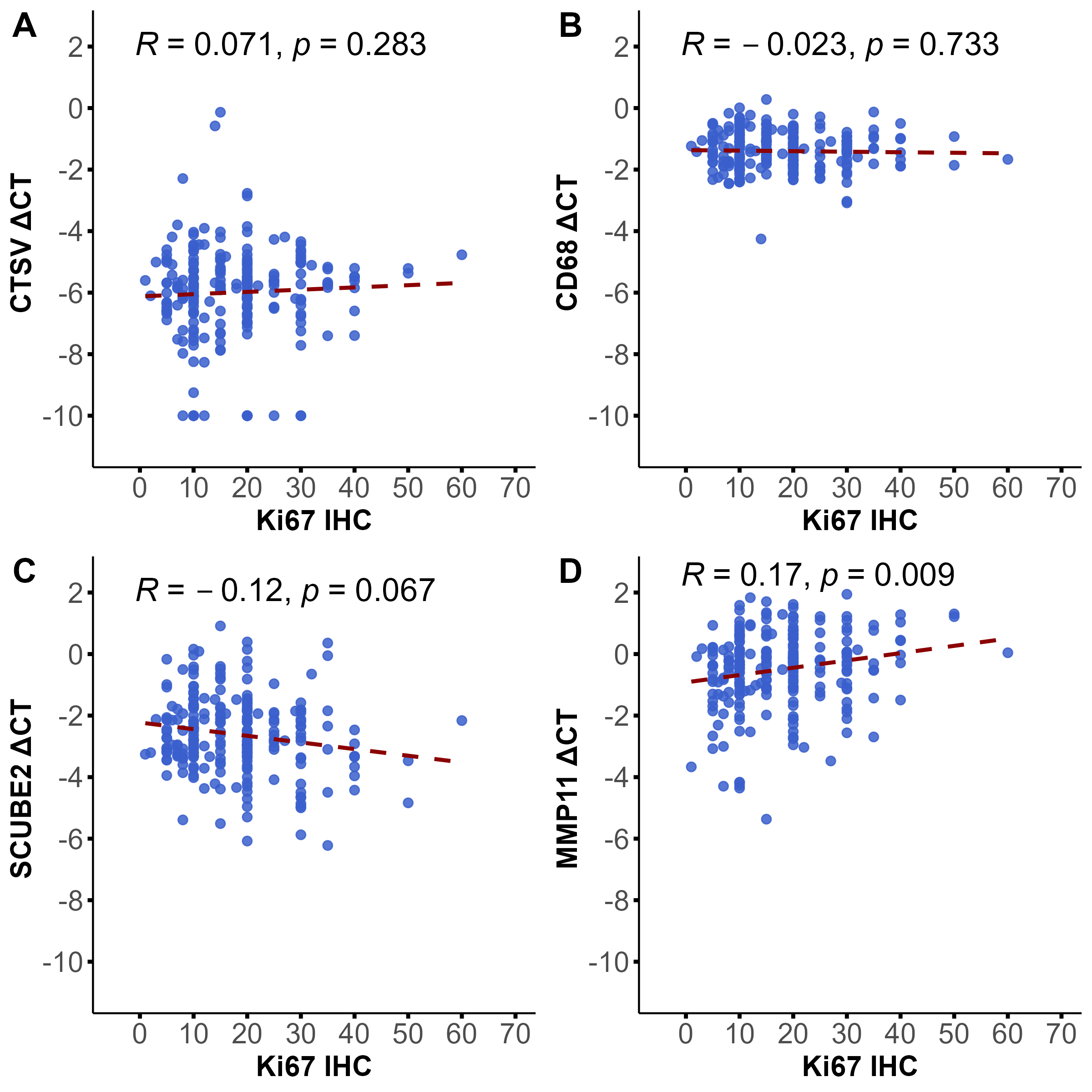

Supplement: Supplementary file 2 — Figure S2: Scatter plots of mRNA ΔCt values and ki67 IHC. A) CTSV mRNA; B) CD68 mRNA. C) SCUBE2 mRNA. D) MMP11 mRNA. Ct: Cycle threshold; IHC: Immunohistochemistry. [file HSR2-9-e72780-s003.png]
