# Supplementary material for: Clinical Significance of Tumor Invasion Gene Profiling in Early‐Stage Hormone Receptor‐Positive Breast Cancer: A Cross‐Sectional Study
Source: Health Sci Rep. 2026 Jul 6;9(7):e72780. doi: 10.1002/hsr2.72780 (PMC13338577; doi:10.1002/hsr2.72780)
Supplement: Supplementary file 3 — Supporting File: hsr272780‐sup‐0003‐Supplementary_Table_1.docx. [file HSR2-9-e72780-s002.docx]

| TaqMan assay | Gene | Protein | Amplicon size |
| --- | --- | --- | --- |
| Hs00968295_m1 | *MMP11* | stromelysin-3 | 60 |
| Hs00952036_m1 | *CTSV* | cathepsin L2 | 72 |
| Hs00154355_m1 | *CD68* | macrosialin | 68 |
| Hs00221277_m1 | *SCUBE2* | signal peptide, CUB and EGF-like domain-containing protein 2 | 64 |
| Hs00942540_m1 | *MYBL2* | myb-related protein B | 71 |
| Hs01060665_g1 | *ACTB* | Actin Beta | 63 |
| Hs00266705_g1 | *GAPDH* | glyceraldehyde-3-phosphate dehydrogenase | 74 |
| Hs00420895_Gh | *RPLP0* | Ribosomal Protein Lateral Stalk Subunit P0 | 76 |
| Hs99999908_m1 | *GUSB* | Glucuronidase Beta | 81 |
| Hs00951083_m1 | *TFRC* | Transferrin Receptor | 66 |

**Supplementary Table 1.** TaqMan assays for target and reference genes
