# Supplementary material for: Clinical Significance of Tumor Invasion Gene Profiling in Early‐Stage Hormone Receptor‐Positive Breast Cancer: A Cross‐Sectional Study
Source: Health Sci Rep. 2026 Jul 6;9(7):e72780. doi: 10.1002/hsr2.72780 (PMC13338577; doi:10.1002/hsr2.72780)

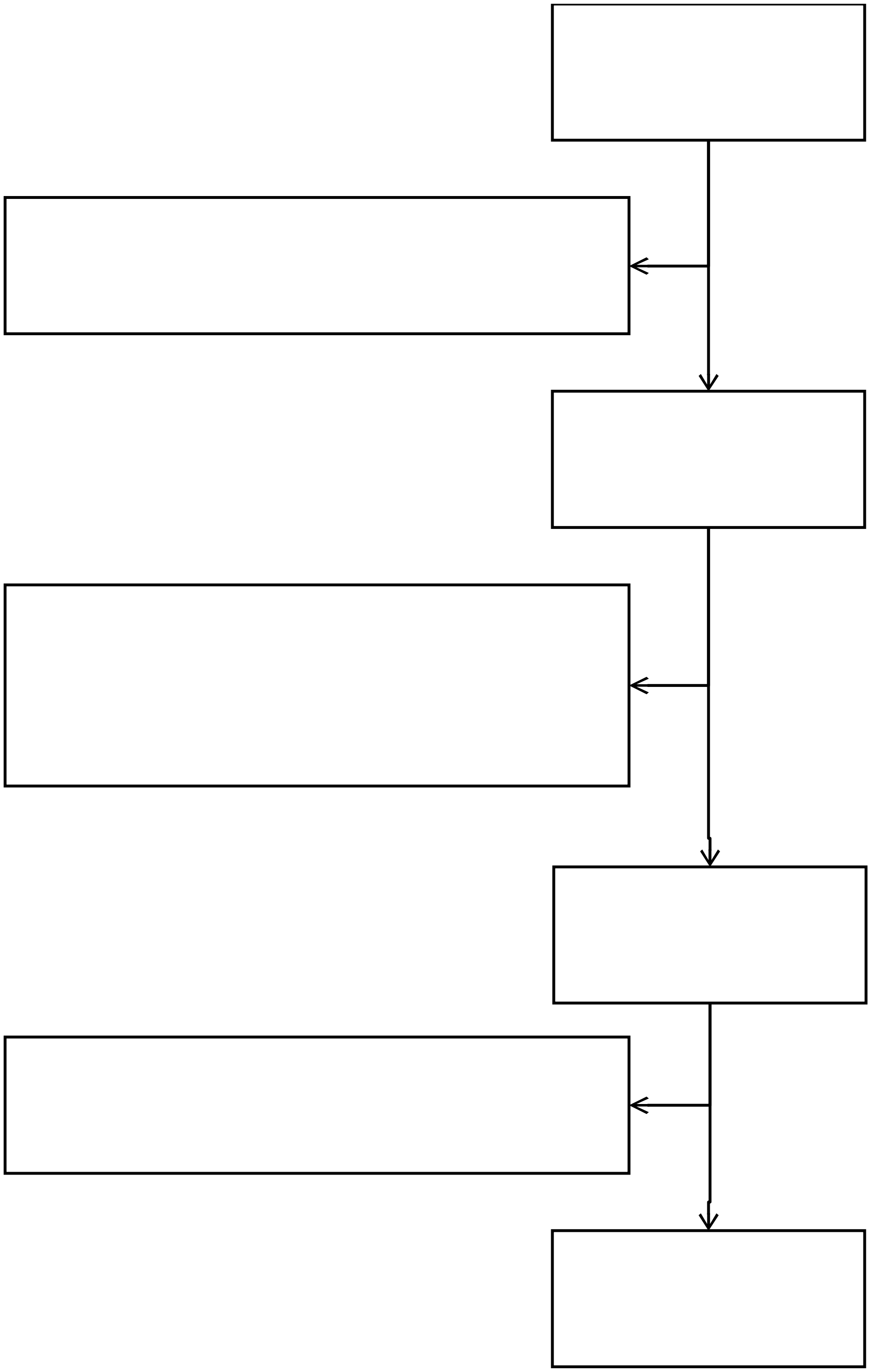

Supplement: Supplementary file 3 [file HSR2-9-e72780-s001.docx]
